# Effect of Hydroxyapatite on the Mechanical Properties and Corrosion Behavior of Mg-Zn-Y Alloy

**DOI:** 10.3390/ma10080855

**Published:** 2017-07-26

**Authors:** Chun Chiu, Chih-Te Lu, Shih-Hsun Chen, Keng-Liang Ou

**Affiliations:** 1 Department of Mechanical Engineering, National Taiwan University of Science and Technology, Taipei 106, Taiwan; cchiu@mail.ntust.edu.tw (C.C.); j62051113@hotmail.com (C.-T.L.); shchen@mail.ntust.edu.tw (S.-H.C.); 2 Department of Dentistry, Taipei Medical University Hospital, Taipei 110, Taiwan; 3 Department of Dentistry, Cathay General Hospital, Taipei 106, Taiwan; 4 Department of Dentistry, Taipei Medical University-Shuang Ho Hospital, New Taipei City 235, Taiwan; 5 3D Global Biotech Inc., New Taipei City 221, Taiwan

**Keywords:** biodegradable materials, magnesium alloys, hydroxyapatite, corrosion

## Abstract

Mg-Zn-Y alloys with a long period stacking ordered (LPSO) phase are potential candidates for biodegradable implants; however, an unfavorable degradation rate has limited their applications. Hydroxyapatite (HA) has been shown to enhance the corrosion resistance of Mg alloys. In this study, Mg_97_Zn_1_Y_2_-0.5 wt% HA composite was synthesized and solution treated at 500 °C for 10 h. The corrosion behavior of the composite was studied by electrochemical and immersion tests, while the mechanical properties were investigated by a tensile test. Addition of HA particles improves the corrosion resistance of Mg_97_Zn_1_Y_2_ alloy without sacrificing tensile strength. The improved corrosion resistance is due to the formation of a compact Ca-P surface layer and a decrease of the volume fraction of the LPSO phase, both resulting from the addition of HA. After solution-treatment, the corrosion resistance of the composite decreases. This is due to the formation of a more extended LPSO phase, which weakens its role as a corrosion barrier in protecting the Mg matrix.

## 1. Introduction

Biomedical metals and biodegradable polymers are widely used as implants. However, medical metals such as stainless steel and titanium alloys can result in stress shielding of the bone and these permanent implants need to be removed by a secondary surgery after healing. Biomedical polymers are degradable but they do not have sufficient mechanical strength. As a result, application of these materials is limited and new biodegradable materials are needed. The density and elastic modulus of Mg alloys are 1.74–2.0 g/cm^3^ and 41–45 GPa, which are well matched with those of human bone. Mg is non-toxic to the human body and is degradable in the body fluid. The above-mentioned advantages make magnesium alloys potential candidates for degradable biomaterials [1,2,3,4,5,6]. Unfortunately, the corrosion rate of Mg alloys in the human body is too high. The premature loss of mechanical integrity due to the loss of strength and rapid corrosion rate prevent Mg alloys from clinical applications [7,8].

To address the problem, alloying element selection has been applied to improve both strength and corrosion resistance of biodegradable Mg alloys, while surface modification has been commonly adopted to control the corrosion rate. Commercially available Mg alloys, such as AZ31 and AZ91 alloys, have been widely studied for application for biodegradable implants. It has been found that a hydroxyapatite (HA) coating can significantly improve the corrosion resistance of AZ31 and AZ91 alloys in simulated body fluid (SBF) [9,10,11]. However, the alloying element, Al, in these alloys is thought to be a neurotoxic element and might be harmful to the nervous system to some extent [12,13].

Mg alloys with nontoxic elements have been developed for biodegradable implants. For instance, Mg-Ca and Mg-Zn alloys have acceptable biocompatibility; however, mechanical properties of these alloys may not meet the requirement of biodegradable implants [14]. In recent years, Mg-Zn-Y alloys have attracted much attention because of their unique long period stacking ordered (LPSO) structure, which improves both strength and corrosion resistance [13,15,16,17]. Unlike Zn, Y is not an essential element in the human body. The concentration of Y in Mg alloys should be minimized. With a limited daily intake of 4.2 mg/d, yttrium can be treated as a biocompatible element [1]. Therefore, low Y containing Mg-Zn-Y alloy with LPSO having high strength is thought to be biocompatible [13,17]. Zhao et al. studied the mechanical properties of Mg-Zn-Y alloys with low Y content. The results showed that Mg_97_Zn_1_Y_2_ alloy exhibited the best overall mechanical properties [13]. Zhang’s group reported corrosion resistance of LPSO-containing Mg-Zn-Y alloys with different Zn and Y content, and found that Mg_97_Zn_1_Y_2_ showed the lowest corrosion rate [18].

Properties of Mg-Zn-Y alloys can be adjusted by controlling the volume fraction and distribution of LPSO structure in the Mg alloys [18,19,20,21]. With a proper control of volume fraction and distribution of LPSO phase, the Mg-Zn-Y alloy can exhibit a lower biocorrosion rate than those of conventional engineered Mg alloys [16]. Properties of Mg alloys can also be adjusted by making a metal matrix composite based on Mg alloys. With a composition similar to that of a natural bone, hydroxyapatite (HA) is a suitable biocompatible and non-toxic reinforcement in Mg-based composites [22,23]. Witte’s group reported that a metal matrix composite made of AZ91 alloy as a matrix and micro-HA particles as reinforcements showed a better corrosion resistance than AZ91 alloy [24]. The HA particles in the MMC stabilized the corrosion rate and exhibited a more uniform corrosion attack. However, Al in AZ91 alloys is harmful to the human body. Sun’s group studied the in vitro corrosion of Mg-3Zn-0.5Zr-*x*HA composites (*x* = 0, 0.5, 1, 1.5 wt%) [25]. The composite with 1 wt% of nano-HA gave the optimal degradation rate, while the one with 1.5 wt% of nano-HA showed the best overall mechanical properties. Ye et al. synthesized Mg-3.0Zn-0.8Zr-1HA composite and studied its corrosion behavior in SBF [14]. The composite showed better corrosion resistance than that of the Mg-Zn-Zr alloy. The corrosion potential increased from −1.630 V in Mg-Zn-Zr alloy to −1.615 V in the composite. Up to now, the properties of HA-reinforced Mg-Zn-Y composite have rarely been reported. With the improved corrosion resistance brought by the LPSO structure in Mg-Zn-Y alloys, and by the addition of HA in Mg alloys, it is worth investigating the properties of a composite consisting of Mg_97_Zn_1_Y_2_ alloy and HA nanoparticles. Therefore, the mechanical properties and corrosion behavior of the Mg-Zn-Y-HA composite were studied in the present work to investigate the effect of HA on the properties of the synthesized composite.

## 2. Results and Discussion

### 2.1. Microstructural Characterization

**Figure 1:**
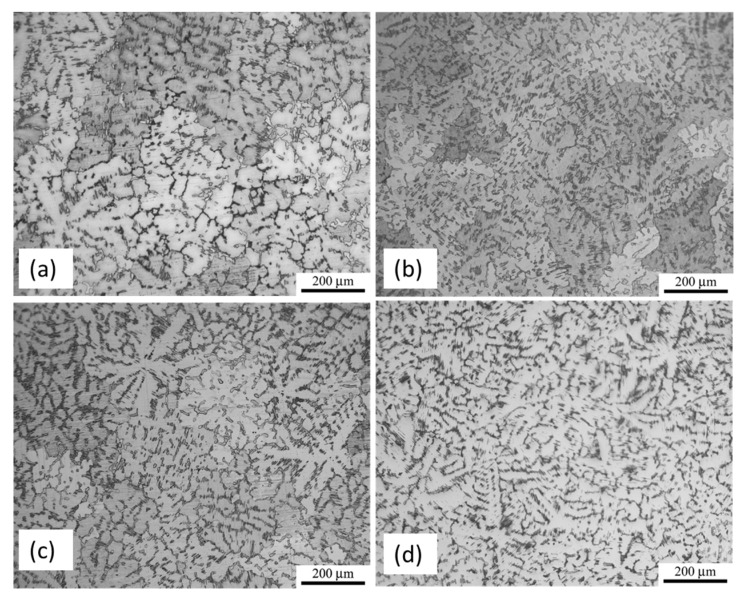
Optical micrographs of (**a**) MgZnY-C; (**b**) HA-C; (**c**) MgZnY-H; and (**d**) HA-T alloy.

Optical micrographs of the as-cast Mg_97_Zn_1_Y_2_ alloy (MgZnY-C), as-cast Mg_97_Zn_1_Y_2_-0.5 wt% HA (HA-C), as well as Mg_97_Zn_1_Y_2_ alloy and Mg_97_Zn_1_Y_2_-0.5 wt% HA solution-treated at 500 **°**C for 10 h (MgZnY-T, and HA-T) are shown in Figure 1. These four alloys consist of primary dendritic matrix and secondary phase distributed along the grain boundary or in the interdendritic regions.

**Figure 2:**
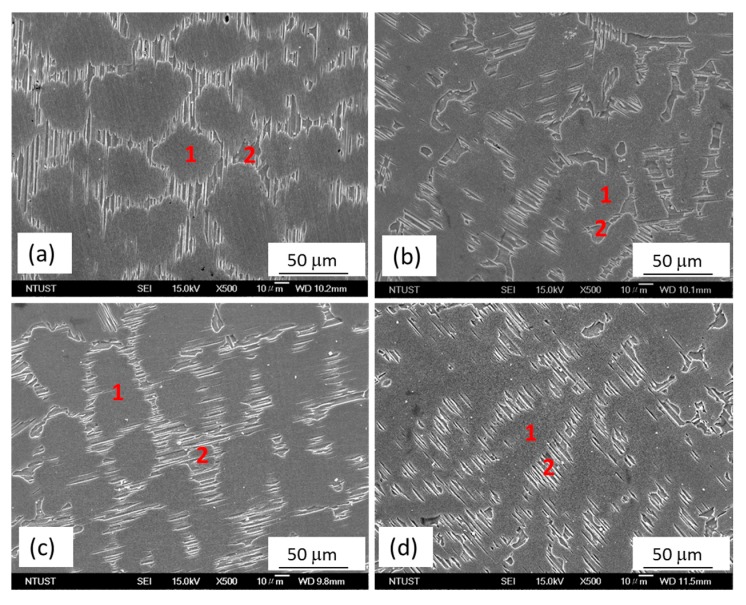
Scanning electron microscopy (SEM) micrographs of (**a**) MgZnY-C; (**b**) HA-C; (**c**) MgZnY-H; and (**d**) HA-T alloys.

**Figure 3:**
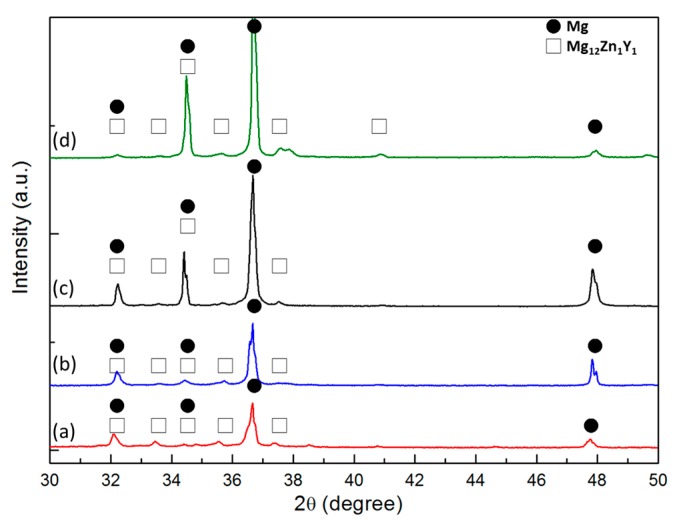
X-ray diffraction (XRD) patterns of (**a**) MgZnY-C; (**b**) HA-C; (**c**) MgZnY-T and (**d**) HA-T alloys.

**Figure 4:**
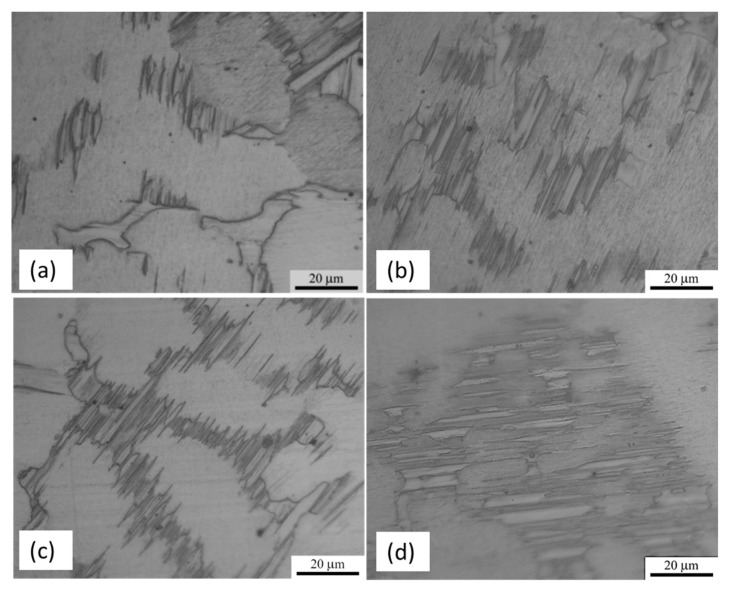
Optical micrographs of as-cast and solution-treated alloys taken at higher magnification showing the growth of secondary phase into the matrix after heat treatment. (**a**) MgZnY-C; (**b**) HA-C; (**c**) MgZnY-T; (**d**) HA-T alloys.

The scanning electron microscopy (SEM) micrographs of the as-cast alloys, MgZnY-C and HA-C, are shown in Figure 2a,b. Energy dispersive spectroscopy (EDS) results given in Table 1 indicate that zone 1 (matrix) is α-Mg solid solution with a relatively low content of Zn and Y, and zone 2 (secondary phase) is a phase with a relatively high content of Zn and Y, which can be assigned to Mg_12_Z_1_Y_1_ phase with a LPSO structure as reported in Refs [13,18,26,27] (Table 1). The corresponding X-ray diffraction (XRD) patterns confirm the presence of the α-Mg and LPSO phase in the MgZnY-C, and HA-C alloy, and reveal that the LPSO phase in the as-cast alloys is a 18R-type structure reported by Yamasaki et al. (Figure 3a,b) [28]. Morphology of the LPSO phase in the MgZnY-C alloy can be described as a semi-continuous network with a small amount of isolated islands (Figure 4a). With the addition of HA, the network breaks down and changes to a discontinuous type (Figure 4b). More isolated islands and lamellar regions are developed for the LPSO phase in the HA-C alloy. The grain size of each alloy is given in Table 2. The grain size decreases from 243 μm in MgZnY-C alloy to 232 μm in HA-C alloy, indicating a slight grain refinement after the addition of HA. The grain refinement is most likely due to the heterogeneous nucleation introduced by HA particles. After addition of HA, the solid/liquid interface energy is reduced, and nuclei can be formed more easily. As compared with grain sizes of the MgZnY-C (243 μm) and HA-C (232 μm) alloys, grain sizes increase in MgZnY-T (254 μm) and HA-T (241 μm) alloys, suggesting a slight grain growth after solution treatment.

**Figure 5:**
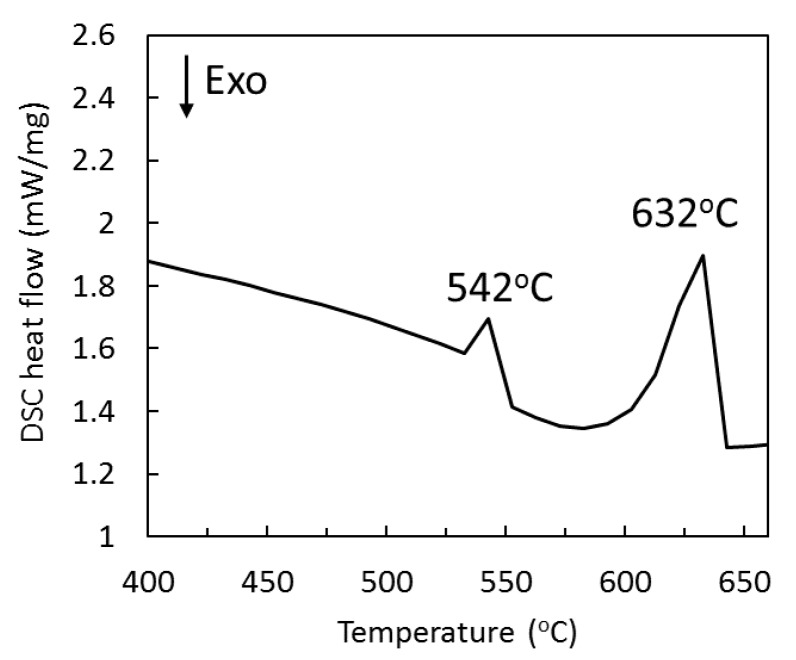
Differential scanning calorimetry (DSC) curve of the MgZnY-C alloy showing phase transformation upon heating.

DSC curve of the MgZnY-C alloy is shown in Figure 5. Two endothermic peaks at 542 **°**C and 632 **°**C can be observed. The secondary peak correlates well with the melting of Mg. Results from EDS and XRD analyses indicate that there is only one secondary phase (LPSO) in MgZnY-C alloy. As a result, the first peak in the differential scanning calorimetry (DSC) curve is due to the dissolution of the LPSO phase, which was also reported by Su et al. and Chen et al. for the differential thermal analysis (DTA) thermogram of Mg-Zn-Y alloys [27,29].

Figure 2c,d show the SEM micrographs of the solution treated alloys, MgZnY-T and HA-T. According to the results of EDS and XRD analysis, the matrix and secondary phase in the MgZnY-T and HA-T alloys are α-Mg and LPSO phase, respectively (Table 1 and Figure 3). After solution treatment, the LPSO phase grows into the matrix. The extension of the LPSO phase further breaks down the network, and the amount of isolated islands and lamellar regions also increase (Figure 4c,d). Chen et al. reported the growth of the LPSO phase in Mg_97_Zn_1_Y_2_ alloys after heating at 540 **°**C for 10 h [29]. The extended LPSO phase reached the center of the grain or interdendritic region. However, the extension of the LPSO phase in the MgZnY-T and HA-T alloys in the present work is limited to the vicinity of the grain boundary or the interdendritic regions. The extension of the LPSO phase is due to the diffusion of Y and Zn, and is enhanced when the treatment time or temperature increases. With a lower heating temperature used in the heat-treated alloys in the present work, the extension of the LPSO phase is limited.

The existence of 18R-LPSO phase in the as-cast alloys was confirmed by XRD analysis in the current study. Regarding the type of LPSO structure in the solution treated alloys, the 18R-type LPSO phase can still be observed in the XRD patterns of the MgZnY-T and HA-T alloys. However, it has been reported that transformation of 18R-LPSO phase to 14H-type LPSO phase occurs when the annealing temperature is higher than 350 **°**C [30]. Therefore, the presence of 14H-LPSO phase in the solution treated alloys cannot be ruled out. Previous studies have shown that diffraction peaks of 14H-LPSO phase overlap with those of Mg phase. As a result, with the presence of 14H-LPSO phase, the peak intensities increase sharply [30,31]. As compared with the XRD patterns of the as-cast alloys, a sharp increase of intensities of Mg peaks in the solution treated alloys can be observed, indicating the peaks corresponding to 14H-type phase overlap with those of Mg (Figure 3). Thus, both of 18R and 14H type of LPSO phase exist in the solution treated alloys.

### 2.2. Mechanical Properties

The volume fraction of the phases, yield strength (YS), ultimate tensile strength (UTS), and elongation of the as-cast and solution treated alloys are given in Table 2. The volume fraction of the LPSO phase decreases from 24.9% in the MgZnY-C alloy to 17.7% in the HA-C alloy. YS and UTS of the MgZnY-C alloy are 126 and 172 MPa, respectively, while the YS and UTS of the HA-C alloy are 117 and 161 MPa, respectively. With the addition of HA, YS, and UTS of the MgZnY alloy decrease slightly. According to the Hall-Patch equation, the strength increases as the grain size decreases. The grain refinement in the HA-C alloy is expected to increase the strength as compared with that of MgZnY-C alloy. The hardness value of the LPSO phase (118 HV) is higher than that of α-Mg (73 HV) in the MgZnY-C alloy. As a result, the role of the LPSO phase in tested alloys can be treated as reinforcement while α-Mg can be seen as the matrix in the alloy. The YS and UTS are expected to decrease with the decrease in the volume fraction of the LPSO phase. However, the differences of the YS and UTS values between the MgZnY-C and HA-C alloys are small even though the volume fraction of the LPSO phase is lowered in the HA-C alloy. This is most likely due to the grain size strengthening in the HA-C alloy, which compensates the negative effect resulting from the decrease of LPSO phase in the HA-C alloy. Thus, no sharp decrement of mechanical properties is observed although the volume fraction of LPSO phase is lowered after the addition of HA.

YS and UTS of the MgZnY-T alloy are 108 and 179 MPa, respectively, while the YS and UTS of the HA-T alloy are 109 and 163 MPa, respectively. YS values of the alloys decrease slightly after solution treatment (Table 2). The effect of solution-treatment on the mechanical properties can be discussed based on grain size, and volume fraction, morphology, distribution and type of LPSO phase. A slight grain growth is observed in the solution-treated alloys, which has a negative effect on the strength.

Zhang et al. reported mechanical properties of Mg-Gd-Zn-Zr (GZ51K) alloy [19]. After T4-treatment (heating at 520 °C for 12 h and cooling in room-temperature water), most of the LPSO phase in the GZ51K alloy dissolved into the α-Mg matrix and resulted in lower YS and UTS. Under the heat treatment parameters with a heating temperature of 500 **°**C and time of 10 h, the LPSO phase is still observed in solution treated MgZnY alloys studied in the present work, which suggests that the dissolution temperatures of the LPSO phases in the Mg-Zn-Y and Mg-Gd-Zn-Zr alloys are different. Volume fractions of LPSO phases in the solution-treated alloys and as-cast alloys do not differentiate much. Thus, the impact of volume fraction on the strength is very small.

Chen et al. studied the effect of morphology change on the mechanical properties of heat-treated MgZnY alloys [26]. It is reported that the fully-extended LPSO phase, which has a lamellar structure through the entire matrix region, is beneficial for the improvement of YS and UTS. However, for the solution-treated alloys in the current study, the extension of the LPSO phase is limited to the vicinity of the LPSO phase region, plus the fact that the volume fractions of the LPSO phases are very similar to those in the as-cast alloys. Thus, the influence of the morphology change brought by heat treatment on the strength is less effective in the present work.

As mentioned earlier, the 18R-LPSO phase distributing along the grain boundary or interdendritic region grows toward the α-Mg region and transforms to 14H-LPSO phase having a lamellar structure after solution treatment. The Vickers hardness values of the α-Mg and LPSO phase are given in Table 3. It can be seen that hardness values of α-Mg in the four alloys are very similar. As a result, neither the addition of HA nor the solution treatment affects Vickers hardness of α-Mg, confirming that the distribution of the LPSO phase is restricted to the region near the grain boundary or interdendritic region. On the contrary, hardness values of the lamellar LPSO phase in the MgZnY-T and HA-T alloys are lower than those in the MgZnY-C and HA-C alloys, suggesting the strengthening effect of the lamellar LPSO phase is lower. The difference in the hardness also implies the transformation of the LPSO phase after solution treatment. With the negative effects brought by grain growth and the lower strengthening effect of the LPSO phase after solution treatment, the YS decreases.

Elongation of the HA-C alloy (9.2%) is very close to that of the MgZnY-C alloy (9%), indicating that addition of HA has a very small influence on the elongation of MgZnY alloy. However, elongation of the as-cast alloy is enhanced after solution treatment (14% for MgZnY-T and 12% for HA-T). The improved elongation after solution treatment is due to the transformation of 18R to 14H LPSO. Compared to 18R-LPSO, the 14H-LPSO has a lower hardness, which makes the dislocations glide and matrix deformation more easily.

**Figure 6:**
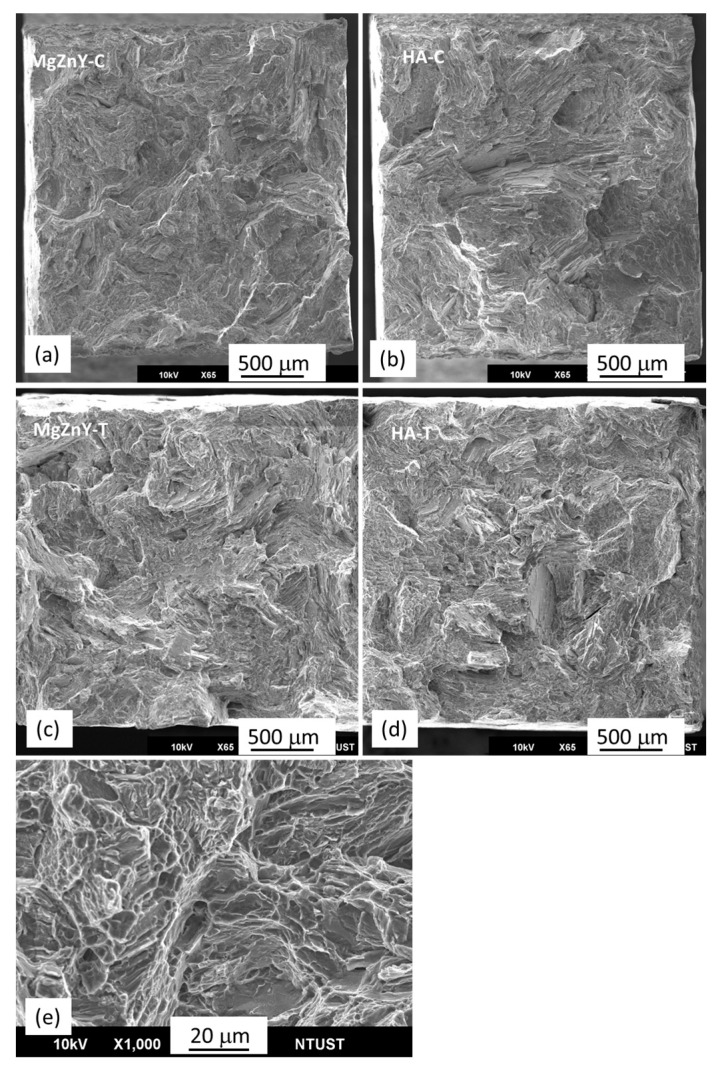
Fracture morphologies of (**a**) MgZnY-C; (**b**) HA-C; (**c**) MgZnY-T, and (**d**) HA-T alloys; (**e**) fracture morphology of solution treated alloys shows dimples.

The fracture morphologies of the MgZnY-C and HA-C alloys show brittle fracture with cleavage planes and tearing ridges (Figure 6a,b), suggesting that the addition of HA has not changed the fracture mechanism. The similarity of the fracture morphology between the MgZnY-C and HA-C alloys agrees well with the results that no significant changes in YS and UTS between the MgZnY-C and HA-C alloys are observed. Similar fracture features are also reported for as-cast Mg-Zn-Y alloys [32]. The fracture morphologies of the MgZnY-T and HA-T alloys show mainly cleavage planes and tearing ridges (Figure 6c,d). However, in addition to these features, dimples can be observed in the solution treated alloys (Figure 6e), implying improved elongation in the solution treated alloys.

### 2.3. Corrosion Behavior

**Figure 7:**
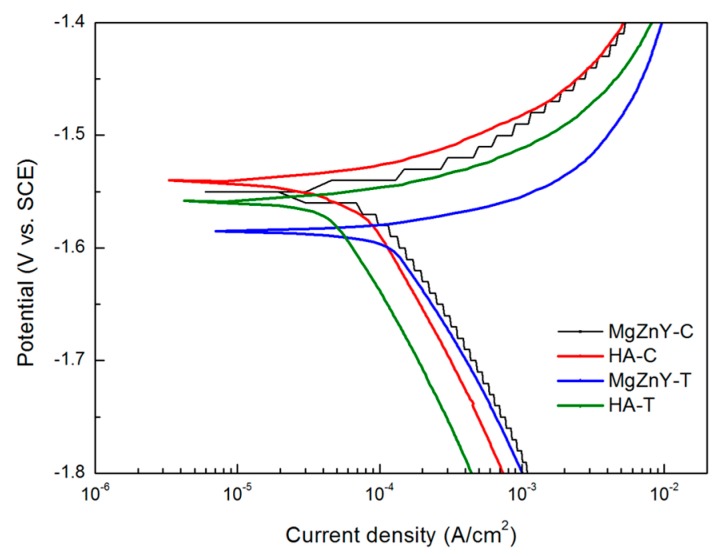
Potentiodynamic polarization curves of MgZnY-C, HA-C, MgZnY-T, and HA-T alloys measured in simulated body fluid (SBF) solution.

The corrosion behavior of the tested alloys is compared by measuring potentiodynamic polarization curves. The polarization curves of samples measured in SBF are shown in Figure 7 and the results are summarized in Table 4. There is no significant difference between the corrosion potential (E_corr_) of the HA-C (−1.55 V) and MgZnY-C alloy (−1.54 V); however, the corrosion current density (I_corr_) of the HA-C (65 μA/cm^2^) is 55% of that of the MgZnY-C alloy (119 μA/cm^2^), indicating HA-C has a better corrosion resistance. Overall, the current densities of the solution-treated alloys are higher than those of the as-cast alloys, suggesting that corrosion resistance of the alloys is reduced after solution treatment at 500 **°**C.

**Figure 8:**
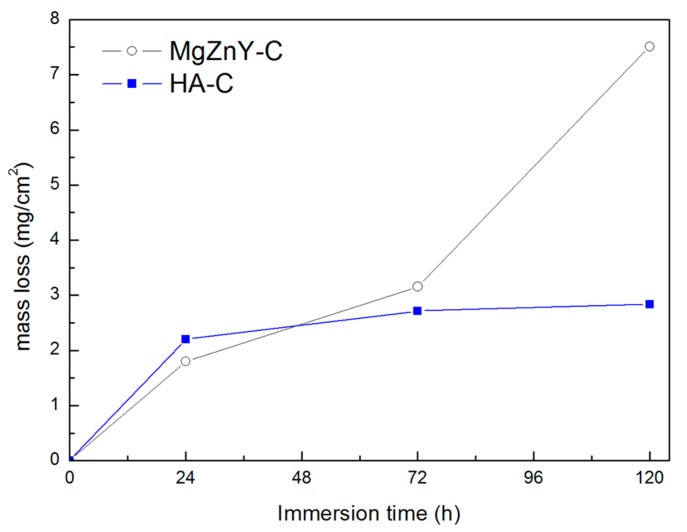
Mass losses of MgZnY-C and HA-C alloys as a function of immersion time.

**Figure 9:**
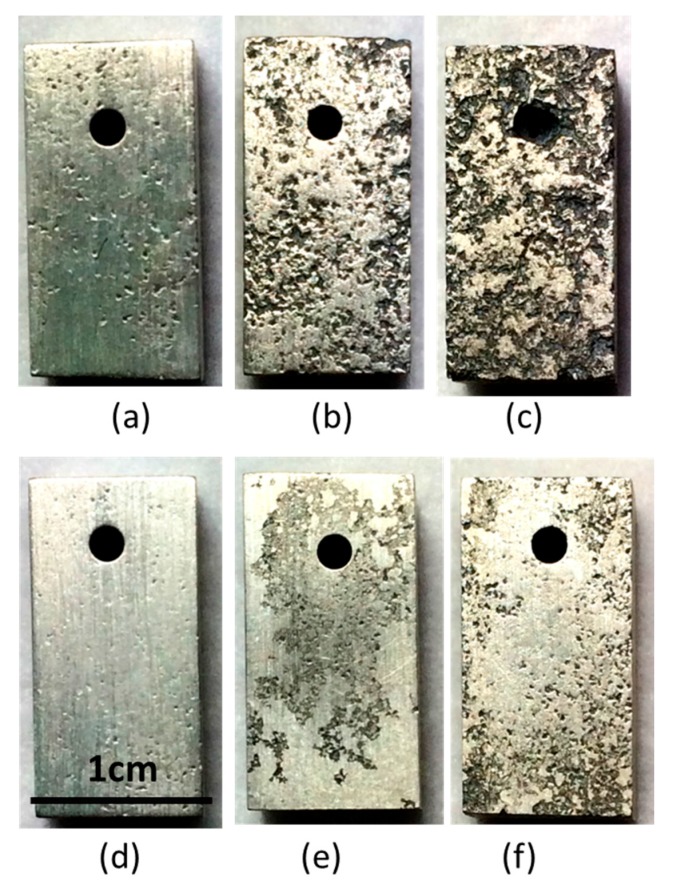
Macro corrosion morphologies of the MgZnY-C alloy immersed in simulated body fluid (SBF) for (**a**) 24 h; (**b**) 72 h; and (**c**) 120 h; and of HA-C alloy immersed in SBF for (**d**) 24 h; (**e**) 72 h; and (**f**) 120 h.

The immersion test was also performed on the as-cast alloys to confirm the corrosion resistance measured by the polarization test. The mass losses of the as-cast alloys immersed for 24, 72, and 120 h are shown in Figure 8. Mass losses of the MgZnY-C and HA-C alloys increase with immersing time; however, the mass loss of the MgZnY-C alloy shows a sharp increase with increasing immersing time, while that of the HA-C alloy shows a mild increase. The evolution of macro corrosion morphologies of the MgZnY-C and HA-C alloys after immersing in SBF for 24, 72, and 120 h are shown in Figure 9. Both of the alloys have pitting corrosion, however, it is clear that the corrosion is more sever in the MgZnY-C alloy. The corrosion rate of the HA-C (1.1 mm/year) is only 38% of that of the MgZnY-C alloy (2.9 mm/year).

**Figure 10:**
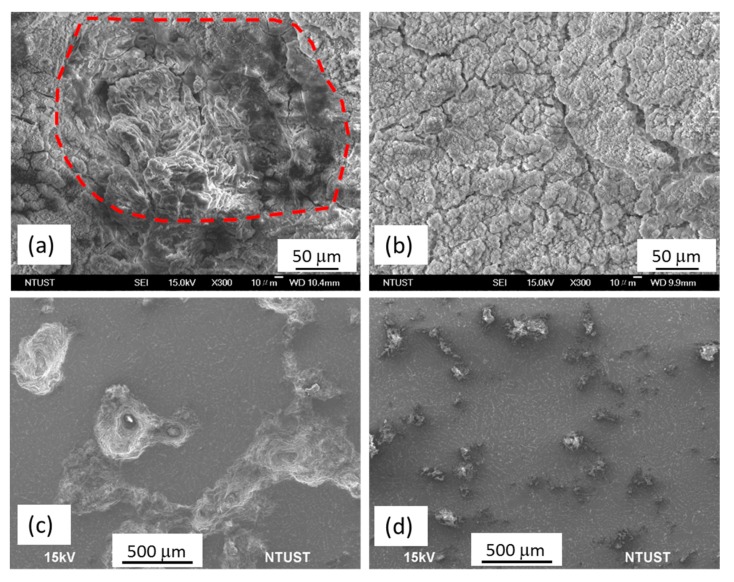
SEM micrographs of as-cast alloys after immersion in SBF: surface morphology of (**a**) MgZnY-C alloy and (**b**) HA-C; corresponding morphology of (**c**) MgZnY-C alloy and (**d**) HA-C alloys after removing corrosion products.

Figure 10 shows the SEM surface morphology of the MgZnY-C and HA-C alloys after immersion in SBF for 120 h. A severely corroded region can be seen on the surface of the MgZnY-C alloy, which indicates that the corrosion layer product is undermined during the immersion test (Figure 10a). After removing the corrosion layer, deeper and wider corrosion pits can be observed (Figure 10c). It is evident that severe corrosion occurs on the surface. Conversely, a more compact corrosion product is observed on the surface of the HA-C alloy (Figure 10b), and small and isolated pitting holes are found after removing the corrosion products (Figure 10d), suggesting slight corrosion. The results from the immersion test agree with the polarization test results that addition of 0.5 wt% HA improves the corrosion resistance of Mg_97_Zn_1_Y_2_ alloy.

It has been reported that corrosion reactions of Mg alloys immersed in a neutral aqueous solution proceed by the following reactions [33,34,35]:

Mg → Mg^2+^ + 2e**^−^** (1)

2H_2_O + 2e**^−^** → H^2+^ + 2OH**^−^** (2)

Mg^2+^ + 2OH**^−^** → Mg(OH)_2_ (3)

In addition, the following reactions probably occur on the surface of Mg alloy when it is immersed in SBF, which contains Cl^−^, Ca^2+^, and PO_4_^3**−**^ [33,35]:

Mg^2+^ + 2Cl**^−^** → MgCl_2_ (4)

*x*Ca + *y*PO_4_^3**−**^ → Ca*_x_*(PO_4_)*_y_* (5)

*x*Ca^2+^ + *y*Mg^2+^ + *z*PO_4_^3**−**^ → Ca*_x_*Mg*_y_*(PO_4_)*_z_* (6)

According to the above equations, the corrosion of Mg_97_Zn_1_Y_2_ alloy immersed in SBF could be addressed as follows: at the initial stage of the immersion test, Mg(OH)_2_ layers are formed on the surface of the sample. After a prolonged immersion time in SBF, Cl**^−^**, CO_3_^2**−**^, PO_4_^3**−**^, OH**^−^**, and Ca^2+^ would diffuse to the surface area. The Cl**^−^** would react with Mg(OH)_2_ and form easily soluble MgCl_2_, resulting in deterioration of the Mg(OH)_2_ layer, which acts as a surface protective layer. Thereafter, Mg^2+^ and/or Ca^2+^ ions would form a new corrosion layer (Equations (5) and (6)). The new corrosion layer containing Ca and P could act as a protection layer on the surface of the Mg_97_Zn_1_Y_2_ alloy if it is intact.

It has been shown that corrosion resistance of Mg alloy could be enhanced by addition of HA particles. Liu et al. studied the corrosion behavior of Mg-4 wt% Zn-*x*HA (*x* = 20% and 40%) composite immersed in SBF [36]. From EDS analysis of the surface layer of the tested composites after the immersion test, it is reported that HA particles have stronger tendency to adsorb Ca^2+^ and PO_4_^3**−**^ ions and improve the integrity of the surface protection layer. The new Ca-P layer would protect the composite from pitting and improve the corrosion resistance. With the increase of HA, the corrosion resistance is further increased. Ye et al. studied the corrosion resistance of Mg-3 wt% Zn-0.8 wt% Zr-1 wt% HA [14]. The observed low corrosion rate is related to the compact Ca-P layer on the surface of the alloy, which is induced by the Ca^2+^ and PO_4_^3**−**^ ions adsorbed by HA particles.

**Figure 11:**
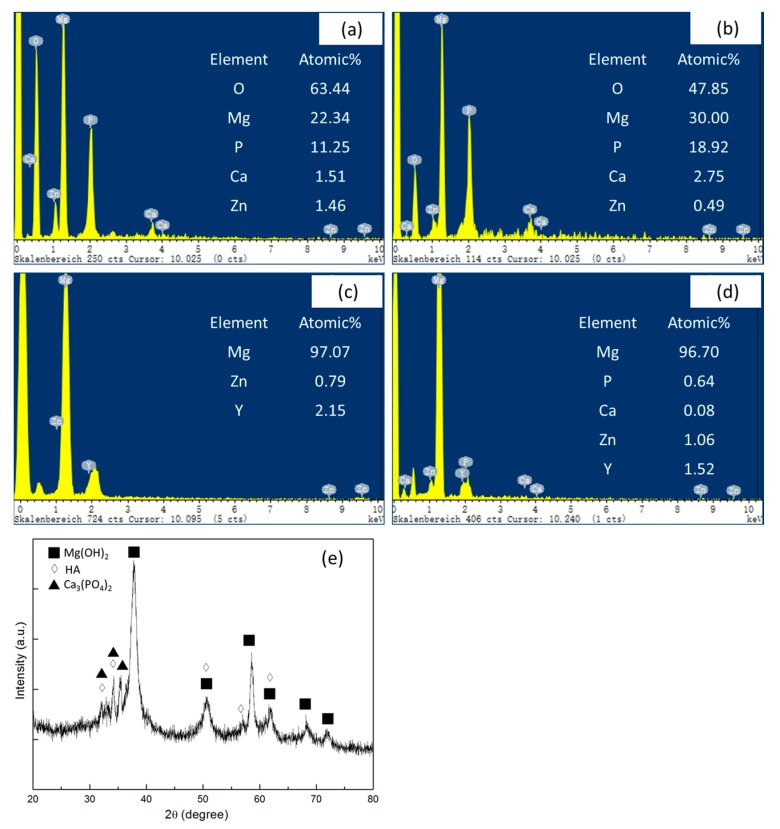
Energy dispersive spectroscopy (EDS) spectrums of surface corrosion layer in (**a**) MgZnY-C alloy and (**b**) HA-C alloys; EDS spectrums of the (**c**) MgZnY-C alloy and (**d**) HA-C alloy before immersion test; (**e**) X-ray diffraction (XRD) pattern of the surface corrosion layer in HA-C alloy.

In the present study, EDS patterns of the surface layer of the MgZnY-C and HA-C alloys after immersing in SBF for 120 h are shown in Figure 11a,b. Compared with the EDS results of the alloys before immersion (Figure 11c,d), the relative intensities of Ca and P increase in the alloys after immersion, suggesting that Ca^2+^ and PO_4_^3**−**^ ions diffuse to the surface and form Ca-P compound. XRD analysis of the surface layer of the HA-C alloy reveals that the Ca-P compound consists of HA and Ca_3_(PO_4_)_2_ (Figure 11e). The amounts of Ca and P in the surface corrosion layer of HA-C alloy are higher than those in MgZnY-C alloy. Thus, the adsorption of Ca^2+^ and PO_4_^3**−**^ ions induced by HA is enhanced by the presence of HA and the integrity of the surface corrosion layer is improved. As a result, inferior corrosion resistance is observed in the HA-C alloy.

**Figure 12:**
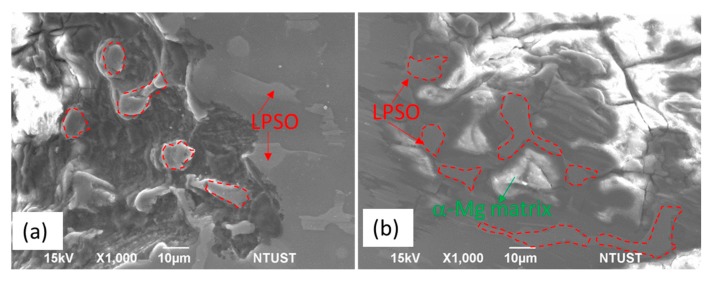
SEM micrograph showing surface near corrosion pits in (**a**) MgZnY-C alloy and (**b**) HA-C alloys.

In addition to HA, the LPSO phase also plays an important role in the corrosion behavior of the Mg alloys [17,18,19,20]. The corrosion surfaces near corrosion pits in the MgZnY-C and HA-C alloys are shown in Figure 12. EDS analysis reveals that the composition of the isolated island inside the pit in the MgZnY-C alloy is Mg-4.7 at% Zn-9.1 at% Y, having similar chemical composition to the LPSO phase in the non-corroded region (Figure 12a). This indicates that the islands are LPSO phases, and the LPSO phase has a higher corrosion resistance than that of α-Mg matrix. Similar results are also found in the HA-C alloys. As shown in Figure 12b, the α-Mg matrix is under more serous corrosion. The difference between the composition of the LPSO phase and the α-Mg matrix results in potential difference, and micro-galvanic corrosion is observed in both of the MgZnY-C and HA-C alloys. In the galvanic corrosion, the LPSO phase acts as a micro-cathode and α-Mg matrix acts as a micro-anode. The initial corrosion starts at the interface of the LPSO phase and α-Mg matrix. Similar corrosion behavior was also reported for Mg-Zn-Gd-Zr alloys and Mg-Y-Er-Zn alloys containing LPSO phase [20,27].

The volume fraction, distribution, morphology, and type of LPSO phase affect the corrosion resistance of the tested alloys in the present study. The LPSO phase with a morphology of a continuous network in the grain boundary region can act as a corrosion barrier to protect the matrix. On the other contrary, when the LPSO phase is composed of isolated islands and a lamellar region, the hindering effect is lost. The decrease of the LPSO phase would improve the corrosion resistance since the microgalvanic corrosion is weaken. In the present study, better corrosion resistance is observed when 0.5 wt% of HA is added into HA-C alloy. In the HA-C alloy, the LPSO phase distributes discontinuously in the grain boundary or interdendritic region. The discontinuously-distributed LPSO phase has a less hindering effect on protecting the α-Mg matrix, and is expected to be detrimental to corrosion resistance. On the other hand, the volume fraction of the LPSO phase decreases in the HA-C alloy, indicating that less micro-cathodes would form and stabilize the corrosion rate. When considering both the negative effect of morphology and the positive effect of the volume fraction of the LPSO phase on the corrosion resistance of HA-C alloys, the latter effect prevails.

There is no huge difference between the volume fraction of the LPSO phase in the solution treated alloys and the as-cast alloys; however, morphology and type of LPSO phase change after solution treatment. A certain portion of the 18R-LPSO phase transforms to 14H-LPSO phase after solution treatment at 500 **°**C. In the solution-treated alloys, the 18R-LPSO phase distributes along the grain boundary or interdendritic region, while the 14H-LPSO phase having a lamellar structure is found in the vicinity of the grain boundary or interdendritic region. Peng et al. reported the effect of different LPSO phase on the corrosion behavior of Mg-Dy-Zn alloy. It was found that a fine 14H-LPSO lamellar structure in grain interior is more effective in improving corrosion resistance than a 18R-LPSO phase in the grain boundary [34]. However, for the solution-treated alloys, such as MgZnY-T and HA-T alloys, the 14H-lamellar structure is only found in the vicinity of the grain boundary or interdendritic region, and is not fully extended through the α-Mg region. With this kind of distribution, the protecting effect is not as effective as the fully-extended one in the grain interior. Therefore, the degradation of corrosion resistance in the solution-treated alloys is due to the formation of a more extended LPSO phase growing into the matrix region, which has less of a hindering effect on protecting the corrosion of the α-Mg matrix.

## 3. Materials and Methods

Mg_97_Zn_1_Y_2_ alloy (at%) was prepared by melting commercial pure Mg (99.98 wt%), Zn (99.9 wt%), and Y (99.9 wt%) ingots in an electric resistant furnace under the protection of a gas mixture (99% CO_2_ and 1% SF_6_) at 750 **°**C. After mechanical stirring, the molten alloy was poured into a steel mold preheated at 200 **°**C and cooled in air. A composite based on Mg-Zn-Y alloys was produced by melting a mixture of Mg_97_Zn_1_Y_2_ alloy and 0.5 wt% of HA nanoparticles (particle size <100 nm) using the same parameters. The as-cast Mg_97_Zn_1_Y_2_ alloy and composite were solution treated at 500 **°**C for 10 h and quenched into water. The chemical compositions of the samples were determined by inductively coupled plasma mass spectrometry (ICP-MS) and results are given in Table 5. The existence of HA in the composite was also confirmed by element mapping (Figure S1).

Microstructural characterization of the samples was conducted with an optical microscope (OM) and a field-emission scanning electron microscope (FE-SEM) equipped with Energy Dispersive Spectroscopy (EDS). The volume fractions of phases in the samples were measured using ImageJ software. Five random locations of each alloy were analyzed. The constituent phases in the samples were examined by X-ray diffraction (XRD) using Cu Kα radiation with the scan range of 20 to 80**°**. The data were collected with a step size of 0.02**°** and time of 0.5 s. LPSO phase was identified using the XRD pattern of as-cast Mg_97_Zn_1_Y_2_ alloy reported by Yamasaki et al. [28]. Room-temperature tensile test was performed using a universal material testing machine. For each condition, three rectangular samples with the gauge size of 20 mm × 5 mm × 3 mm were tested. The fracture morphology was observed using FE-SEM. Vickers microhardness tests were performed using load of 100 g at 15 s dwell time. At least ten readings were made from which the mean value was calculated. Differential Scanning Calorimetry (DSC) analysis of the as-cast sample was performed under an argon flow rate of 150 mL/min with a heating rate of 5 **°**C/min to study the phase transformation.

Corrosion performance was analyzed by the electrochemical test and immersion test. Potentiodynamic polarization measurements were conducted in simulated body fluid (SBF) at 37 **°**C. A three-electrode cell with a platinum as counter electrode, a saturated calomel electrode as reference electrode, and the sample with an exposed surface area of 1 cm^2^ as a working electrode were used. Prior to the test, the electrode was left under open-circuit conditions for 1800 s until a steady free corrosion value was recorded. The tests were performed from −2 V to 2 V with a scan rate of 2 mV/s. The corrosion potential (E_corr_) and corrosion current density (I_corr_) were determined from the Tafel analysis.

Immersion tests were conducted in SBF at 37 **°**C for 120 h. Approximately 60 mL SBF per cm^2^ specimen surface was used during the test and the SBF was renewed every 24 h to keep a relatively stable pH value. Surfaces of rectangular samples with a dimension of 10 mm × 10 mm × 20 mm were ground with SiC sandpaper up to 2500 grit, then ultrasonically cleaned in alcohol and dried. After immersion periods of 24, 72, and 120 h, the samples were removed from the solution, cleaned and dried. The corrosion surfaces of samples were observed using a digital camera and an SEM. The composition of corrosion products was analyzed by EDS. Thereafter, the corrosion products on the samples were removed and the masses of samples were recorded. The corrosion rates were calculated by the mass loss according to the following formula [33]:

(7) $$\mathrm{CR}\;\left(\mathrm{mm}/\mathrm{year}\right)=\;\frac{8.76\times 10^{4}\times \Delta m}{A\times t\times \rho }$$

where CR is the corrosion rate, Δ*m* is the mass loss in g, *A* is the surface area of the sample in cm^2^, *t* is the immersion time in h, and *ρ* is the density of the sample in g/cm^3^.

## 4. Conclusions

The Mg_97_Zn_1_Y_2_-0.5 wt% HA composite was cast and solution-treated at 500 °C. The microstructures of the as-cast and solution-treated alloys mainly consist of α-Mg matrix and LPSO phase distributed discontinuously along the grain boundary or in the interdendritic regions. With the addition of HA and heat treatment, the LPSO phase grows into the α-Mg matrix and more lamellar regions are formed. In contrast to the Mg_97_Zn_1_Y_2_ alloy, the composite presents better corrosion resistance with the addition of HA particles. In the meantime, no decrement in mechanical properties is observed. The improvement of corrosion resistance is due to the formation of the compact Ca-P surface layer. Under the circumstance that the distribution of the LPSO phase is similar in both the MgZnY and HA-containing MgZnY alloys, the decrease of the volume fraction of the LPSO phase also enhances the corrosion resistance. In comparison to the as-cast composite, the solution-treated composite shows a faster corrosion rate, which is due to the formation of a more extended LPSO phase. The strength of the composite does not improve after solution treatment.

## Figures and Tables

**Table 1:** Chemical composition of phases in the samples (at%).

Element	Sample							
	MgZnY-C		MgZnY-T		HA-C		HA-T	
	1	2	1	2	1	2	1	2
Mg	97.9	87.8	97.4	86.9	97.6	88.7	97.9	87.4
Zn	1.3	5.0	1.2	5.9	1.0	5.1	0.7	5.1
Y	0.8	7.2	1.4	7.1	1.4	6.2	1.4	7.4

**Table 2:** Volume fraction of phases, grain size, Vickers hardness, and tensile properties of the tested alloys. Yield strength (YS), ultimate tensile strength (UTS), long period stacking ordered (LPSO).

Sample	α-Mg (%)	LPSO (%)	Grain Size (μm)	YS (MPa)	UTS (MPa)	Elongation (%)
MgZnY-C	75.1	24.9	243 ± 10	126 ± 13	172 ± 9	9.0 ± 0.6
MgZnY-T	77.8	22.2	254 ± 16	108 ± 6	179 ± 3	14.0 ± 2.4
HA-C	82.3	17.7	232 ± 19	117 ± 2	161 ± 2	9.2 ± 0.2
HA-T	82.2	17.8	241 ± 7	109 ± 9	163 ± 18	12.0 ± 5.6

**Table 3:** Vickers hardness (HV) of the the α-Mg and LPSO phases in the tested alloys.

Sample	α-Mg	LPSO
MgZnY-C	73 ± 3	118 ± 6 (18R)
MgZnY-T	72 ± 3	109 ± 3 (18R + 14H)
HA-C	76 ± 3	120 ± 5 (18R)
HA-T	75 ± 4	107 ± 6 (18R + 14H)

**Table 4:** Corrosion potential (E_corr_) and corrosion current density (I_corr_) measured from the polarization curves, and corrosion rate obtained from the immersion test.

Sample	E_corr_ (V)	I_corr_ (μA/cm^2^)	Corrosion Rate (mm/year)
MgZnY-C	−1.54	118.63	2.93
MgZnY-T	−1.57	162.67	-
HA-C	−1.55	65.00	1.11
HA-T	−1.58	76.17	-

**Table 5:** Nominal and analyzed compositions of the samples.

Sample	Mg_97_Zn_1_Y_2_ (MgZnY-C)			Mg_97_Zn_1_Y_2_-0.5 wt% HA (HA-C)			
	Mg	Zn	Y	Mg	Zn	Y	*n*-HA
Nominal composition (wt%)	90.7	2.4	6.9	90.2	2.4	6.9	0.5
Analyzed composition (wt%)	91.7	1.9	6.4	90.4	2.1	7.1	0.4

## Supplementary Materials

The following are available online at www.mdpi.com/1996-1944/10/8/855/s1, Figure S1: SEM and element mapping.
