# Supplementary material for: Effect of Hydroxyapatite on the Mechanical Properties and Corrosion Behavior of Mg-Zn-Y Alloy
Source: Materials (Basel). 2017 Jul 26;10(8):855. doi: 10.3390/ma10080855 (PMC5578221; doi:10.3390/ma10080855)
Supplement: Supplementary file 1 [file materials-10-00855-s001.pdf]

Supplementary Materials: effect of hydroxyapatite on the mechanical properties and corrosion behavior of Mg-Zn-Y alloy

Chun Chiu[1], Chih-Te Lu[1], Shih-Hsun Chen[1], Keng-Liang Ou[2,3,4,5*]

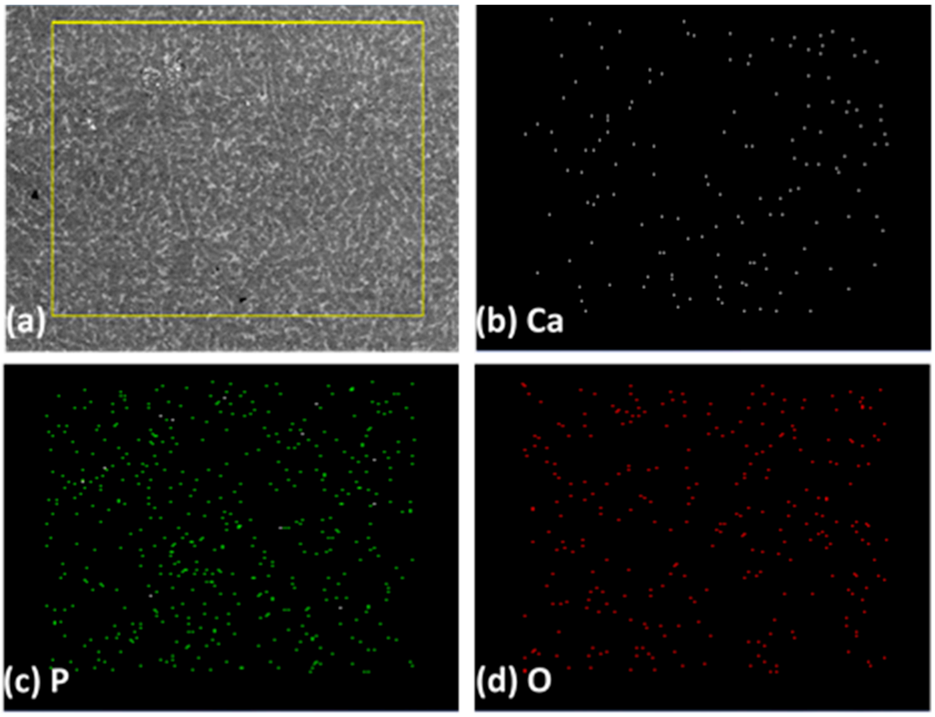

Figure S1 (a) SEM micrograph of HA-C alloy ($Mg_{97}Zn_1Y_2$-0.5wt% HA), and Element mapping of (b) Ca element, (c) P element, and (d) O element in HA-C (SEM and element mapping).
